# Supplementary material for: A reconnaissance survey of farmers’ awareness of hypomagnesaemic tetany in UK cattle and sheep farms
Source: PLoS One. 2019 Oct 11;14(10):e0223868. doi: 10.1371/journal.pone.0223868 (PMC6788701; doi:10.1371/journal.pone.0223868)
Supplement: S1 Information — (PDF) [file pone.0223868.s001.pdf]

S Appendix 1. Semi-structured questionnaire and information sheet for interviewees.

Name / Farm ______________ / ______________ Postcode ______________

Phone ______________ Email ______________

Farm enterprises: please fill in the number of each livestock type on your holding

	Dairy	Sucklers	Beef	Sheep	Other (please specify)
Number					

Have you had problems with magnesium tetany/grass staggers? Please circle **Y / N**

If **yes**, how many animals were affected? ______________

If **yes**, which species were affected? ______________

How do you prevent/treat magnesium issues in your livestock? ______________

Do you have your soils tested for nutrient concentration? Please circle **Y / N**

Do you have your animals tested for mineral status? Please circle **Y / N**

Do you have your forage tested for mineral content? Please circle **Y / N**

Are you happy for us to contact you for a further survey? Please circle **Y / N**

If **yes**, would you prefer to be contacted by **email/phone** in **English/Cymraeg** (please circle)

By completing this survey you are agreeing for us to use this data for our research. For further information see attached letter or www.magnesium-network.uk/survey

PLEASE RETAIN THIS FORM

The University of Nottingham
UNITED KINGDOM · CHINA · MALAYSIA

Survey information / Consent
Title of the study: **MAGNET – Preliminary Survey**
Researchers:
Dr Nigel Kendall (nigel.kendall@nottingham.ac.uk) 0115 9516447
Mr Diriba Kumssa (diriba.kumssa1@nottingham.ac.uk)

Purpose of the study:
The overall aim of this project is to understand the causes of grass tetany/ hypomagnesaemia in ruminants in England and Wales. We will ultimately be investigating the role of soil, forage and feed and livestock. We also want to understand current knowledge and views of livestock farmers regarding magnesium issues.
The information from this study will help us to understand the current views and knowledge of stakeholders regarding magnesium in ruminant livestock and this will help us to develop management strategies to reduce the incidence of grass tetany and improve ruminant nutrition in England and Wales.

The project has 3 phases of collection:
A short key detail questionnaire to determine how many farms have magnesium issues, what they do to treat/prevent the issues and what testing is done on farm. This survey will also ask if you are willing to participate in the next stage.
A more detailed survey which will delve much deeper into farm practice, farmer opinion and farm data if available.
On farm mineral audits, where farms will be selected on the basis of previous information supplied and appropriate locations (based on underlying geology and trial logistics) and the farm will be visited for the collection of data and samples to determine the farm's mineral status.

How can you help.
Please fill in the attached survey or complete this survey online (www.magnesium-network.uk/survey).
If you wish you participate in part 2 then please indicate this and you will be contacted. You do not have to participate any further - the preliminary survey data is still valuable to us.

Consent:
Please read the points below, by submitting the survey you will indicate your consent to the preliminary survey only. Any information collected by the researchers:
will be used for this research study
will only be accessed by authorised research colleagues
will be anonymised and treated confidentially in all outputs/reporting
may be used in a report for publication
may be presented at research conferences or meetings
that you can request to see a copy/summary of the completed study
that you can request to see any information provided by you which is written down/kept during the process of data collection.
that you can withdraw your permission after survey submission
If you have any queries regarding this study, please speak to the researcher directly or contact them via e-mail or phone (details above).

PLEASE RETAIN THIS FORM

The University of Nottingham
UNITED KINGDOM · CHINA · MALAYSIA

Survey information / Consent
Title of the study: **MAGNET – Preliminary Survey**
Researchers:
Dr Nigel Kendall (nigel.kendall@nottingham.ac.uk) 0115 9516447
Mr Diriba Kumssa (diriba.kumssa1@nottingham.ac.uk)

Purpose of the study:
The overall aim of this project is to understand the causes of grass tetany/ hypomagnesaemia in ruminants in England and Wales. We will ultimately be investigating the role of soil, forage and feed and livestock. We also want to understand current knowledge and views of livestock farmers regarding magnesium issues.
The information from this study will help us to understand the current views and knowledge of stakeholders regarding magnesium in ruminant livestock and this will help us to develop management strategies to reduce the incidence of grass tetany and improve ruminant nutrition in England and Wales.

The project has 3 phases of collection:
A short key detail questionnaire to determine how many farms have magnesium issues, what they do to treat/prevent the issues and what testing is done on farm. This survey will also ask if you are willing to participate in the next stage.
A more detailed survey which will delve much deeper into farm practice, farmer opinion and farm data if available.
On farm mineral audits, where farms will be selected on the basis of previous information supplied and appropriate locations (based on underlying geology and trial logistics) and the farm will be visited for the collection of data and samples to determine the farm's mineral status.

How can you help.
Please fill in the attached survey or complete this survey online (www.magnesium-network.uk/survey).
If you wish you participate in part 2 then please indicate this and you will be contacted. You do not have to participate any further - the preliminary survey data is still valuable to us.

Consent:
Please read the points below, by submitting the survey you will indicate your consent to the preliminary survey only. Any information collected by the researchers:
will be used for this research study
will only be accessed by authorised research colleagues
will be anonymised and treated confidentially in all outputs/reporting
may be used in a report for publication
may be presented at research conferences or meetings
that you can request to see a copy/summary of the completed study
that you can request to see any information provided by you which is written down/kept during the process of data collection.
that you can withdraw your permission after survey submission
If you have any queries regarding this study, please speak to the researcher directly or contact them via e-mail or phone (details above).
