# Supplementary material for: A reconnaissance survey of farmers’ awareness of hypomagnesaemic tetany in UK cattle and sheep farms
Source: PLoS One. 2019 Oct 11;14(10):e0223868. doi: 10.1371/journal.pone.0223868 (PMC6788701; doi:10.1371/journal.pone.0223868)
Supplement: Attachment — Submitted filename: Response to reviewers.docx [file pone.0223868.s003.docx]

Response to reviewers

We would like to thank the editor and reviewers for their interest in the manuscript. We are grateful for the constructive criticism and comments. Responses to the comments and suggestions are given in Table 1.

Table 1. Response to reviewers.

| **Reviewer comments** | **Authors responses** |
| --- | --- |
| I would also like to see a small paragraph as a bit of advice to farmers. | Practical implications and conclusion sections added to the manuscript. |
| Line 32- There was a highly significant  Line 55- although you define MypoMgT in the abstract, it would also be useful to define it early in the introduction as well, as some people (like me) don’t read the abstract until the end.  Line 61- same is true for defining PHT  Line 65- this is a little unclear, I do wonder if it is meant to read as hypomagnesaemia related conditions were seen in 1-4% of cows ….  Line 70-71- again unclear. I think it should read where magnesium, usually as part of a mineral premix, ….  Line 82- would the reference not be better written as Roderick et al., ?  Line 103- Should read- Prior to completing the survey, farmers were presented with an information sheet……  Line 108- Should read - English and Welsh, in both paper and digital form, and participants….  There is a stray ‘o’ in line 170- not sure what that should be  Line 174 – maybe should read as …’76% had used a similar range…’  Line 265 would the reference not be better written as Roderick et al., ?  Line 274. Sentence break needed …. In this survey. Further research ….  Line 278, comma after the reference may help flow  Line 288, a comma after ‘did not contain Mg,’ may aid flow  Line 307- systems may read better | These were all actioned |
